# Assessment of Referees in Terms of Building a Positive Climate and Responsiveness to the Health, Emotional, and Social Needs of Rugby Players in Competitive Children Sport

**DOI:** 10.3390/ijerph181910086

**Published:** 2021-09-25

**Authors:** Katarzyna Płoszaj, Wiesław Firek, Paulina Ciszewska-Hołda

**Affiliations:** 1 Faculty of Physical Education, Józef Piłsudski University of Physical Education in Warsaw, ul. Marymoncka 34, 00-968 Warsaw, Poland; katarzyna.ploszaj@awf.edu.pl; 2 Institute of Medical Sciences, University of Opole, pl. Kopernika 11a, 45-040 Opole, Poland; paulina.ciszewska-holda@uni.opole.pl

**Keywords:** referee, sport official, rugby, educational practice, positive climate, children sport

## Abstract

One of the key elements of effective sports programs that contribute to a child’s sense of joy and satisfaction in participating in sports is contact with an adult who builds a caring climate deliberately and intentionally. Referees play a special role in providing positive experiences for young athletes. The main aim of this study was to assess the quality of referee–player interactions in terms of building a positive climate during the game and the referee’s responsiveness to the health, emotional, social, and cognitive needs of young rugby players aged 6–12 years. The research was conducted among 23 referees refereeing children’s rugby matches in Poland. The structure observation method and The Referee–Players’ Interaction Assessment Scoring System tool were used in the study. Each referee was observed twice. The referee’s verbal and non-verbal communication was recorded using a GoPro Hero 8 camera and an Ejeas Fbim wireless intercom system. A Wilcoxon signed-rank test was used to examine differences between referees’ ratings on two dimensions. The Mann-Whitney U test was employed to test the significance of differences in the mean scores of referees between groups divided by experience. The referees, in both dimensions (*building positive climate* and *responsiveness to the players’ needs*), scored on a 7-point scale, were rated at an average level of 3.22 (SD = 1.65) and 4.39 (SD = 1.67), respectively, with the *responsiveness* dimension rating being statistically significantly higher than the *positive climate* rating. The results showed that referee experience was not a determinant of the rating of the quality of referee–players’ interactions in both dimensions. From these studies, it was concluded that there is a need to complement the existing training programs for referees with the contents from the field of pedagogy and developmental psychology. This will help them build a positive climate during the game and show how to respond to children’s health, cognitive, emotional, and social needs. Furthermore, developing a referee’s pedagogical skills can improve their relationship with players, which can ultimately translate into a greater sense of job satisfaction. The formulated practical implications can also be transposed to other team sports.

## 1. Introduction

A caring climate is an indispensable part of any relationship [1]. A person who cares for others meets their needs by listening, empathizing, accepting them, and focusing on fostering their development and progress, building mutual respect and trust [2,3]. The results of empirical research show that interactions in the school classroom or other educational environments based on a positive climate, responsiveness of the educator to the needs of the child, and taking into account his or her perspective increase the child’s commitment and motivation to work, improve the quality of relationships with peers and teachers, and shape social attitudes [4,5,6,7,8]. Although the above research referred to the school environment, it is advisable to also consider this approach in the sports environment, where adult actors interact in numerous ways with young athletes [7,9].

Researchers working in the field of children’s and youth sport are constantly looking at how the sporting environment can become more youth-friendly [7,9,10,11]. They stress that the most important thing for children is to enjoy the effort and participate in the game [12,13,14]. They postulate that approaches aimed at building a positive climate, which is seen as the basis or precondition for a young player to perceive a particular environment as encouraging, safe, supportive, and capable of providing them with positive experiences, e.g., being valued and respected, should be introduced at various sports tournaments and games [15,16]. These experiences are key to strengthening a child’s self-esteem, developing their morals, and reducing anxiety [17,18,19]. If children’s and youth sport becomes excessively focused on the competition, sports results, and the evaluation of a player’s performance by other athletes, coaches, referees, and spectators [20], this will perpetuate the trend of children and youth dropping out of sports [21,22,23].

Hellison [24] emphasizes that one of the key elements of effective sports programs that contribute to a child’s sense of joy and satisfaction in participating in sports is contact with an adult who builds a caring and thoughtful climate deliberately and intentionally. Coaches play a special role in providing positive experiences for young athletes. Research has shown that a coach who uses positive reinforcement, provides appropriate feedback, and creates a climate conducive to the completion of tasks and maintaining the autonomy of young players contributes to building friendly relationships with them, the children’s enjoyment and satisfaction with the sport, their greater involvement in sport, and reduced the tendency to drop out of sports [14,25,26,27,28]. Parental involvement in sports competition of children and adolescents and its significance (positive, negative) for practicing sport by young players and their personality development was also studied [29,30,31,32,33]. Relatively little research on building a positive climate has been devoted to the sports referee, who is the only adult on the field during sports competitions. Therefore, his or her attitudes and actions have a significant effect on the emotional state of the players and the atmosphere during the match. In sports such as basketball, handball, football, rugby, boxing, floorball, etc., the referee interacts with the players in numerous ways [34]. If the referee behaves inappropriately e.g., criticizes the players, speaks disrespectfully to them, or ignores questions, the child who was looking forward to participating in the match may become sad, angry, and frustrated. Therefore, the referee’s actions on the field should be conscious and purposeful and correspond to the psychophysical needs of a child at a given age. Płoszaj, Firek, and Czechowski [35] and Andersson [36] pointed out that for the sports field to become a child-friendly educational environment, referees should, in addition to the coach and parents, build a positive climate. They influence not only the behaviors and experiences of players, but also those of coaches, parents, fans, and other sports activists. 

The main aim of this study was to assess the quality of referee–player interactions in terms of building a positive climate during the game and the referee’s responsiveness to the health, emotional, social, and cognitive needs of young rugby players aged 6–12 years. Building a positive climate should be viewed as “the emotional bond between the referee and players, expressing mutual interest, enthusiastic attitudes and joy of contacts”, while responsiveness “reflects the referee responding to the emotional, cognitive, social, and health needs of players” [35] (p. 4). Furthermore, it was decided to identify differences in the assessment of the aforementioned dimensions of the referee’s work and to verify whether refereeing experience was a determinant of these assessments. The following research hypotheses were formulated: Experienced referees have higher levels of interaction quality in both dimensions compared to inexperienced/novice referees. An experienced referee means a person who has refereed children’s matches for more than 1 year, whereas in a novice referee, this time is one year or less. The criterion for the division of the respondents according to the level of experience was arbitrary and resulted from consultation with the Referee Department in the Polish Rugby Union. Learning about the referee’s interactions with the children during the game in the area studied will help indicate those areas that can be improved to make the sporting event a positive experience for the player. The findings can be used to guide the modification of training programs for rugby referees working with children. Improving a referee’s pedagogical skills can improve their relationship with players, which can ultimately translate into a greater sense of job satisfaction. The practical conclusions can also be transposed to other team sports in which the referee influences the course of the game and interacts with children in numerous ways [37]. The results of the research may be of interest to sports associations and organizations working for the benefit of children’s and youth sport.

## 2. Materials and Methods

### 2.1. Participants

The research was conducted among 23 referees refereeing children’s rugby matches in Poland during tournaments organized by the Polish Rugby Union in the following categories: *Mikrus* (boys and girls born in 2012 and younger), *miniżak* (boys and girls born from 2010–2011), and *żak* (boys and girls born in 2009). There were 22 male and 1 female referees in the group studied. The mean age of the participants was 25.26 years (SD = 8.78). The youngest referee was 17 years old, whereas the oldest was 42. The average refereeing experience was 2 years, which is consistent with the common practice of many sports associations to have younger and less experienced referees officiate children’s competitions. Children’s games are approached as preparation for work with older players.

### 2.2. Measures

The structure observation method was used in the study. This method is used when one wants to find out the natural behavior of people and to limit the subjectivity of the researcher and the respondents. This method is particularly useful when respondents are unable or incapable of expressing an opinion on a particular topic themselves [38]. One of the advantages of this method is the direct observation of behavior, as opposed to surveys, which only allow inferring about behavior [39,40]. Structure observation is a technique in which the researcher uses clearly formulated rules of observation and knows well what is being observed and how to record the behavior of each subject. The Referee–Players’ Interaction Assessment Scoring System [35] tool was used in the study. This tool is used to examine the educational interactions of the referee with the players. The theoretical assumptions of this tool include the hypothesis that the referee, in addition to the coach and parents, is an important link in the process of education through sport. The primary mechanism for educational interactions is the referee’s interactions with the players. The educational function of the referee is to provide players with appropriate emotional support, instructional support, and game organization. Since the aim of our study is to explore the referees’ actions in building a positive climate during the game and their responsiveness to the needs of young players, the emotional support section of the R-PIASS tool was used in this study. The method of assessing individual indicators is presented in Table 1. Examples of poor and good quality in referee–player interactions in terms of *building a positive climate* and *responsiveness* are shown in Table 2 and Table 3.

### 2.3. Procedures

The selection of the group was purposive. The referees of matches for children aged 6–12 organized by the Polish Rugby Union were selected. The research was conducted in cooperation with the Polish Rugby Union, which was informed of the assumptions of the research project. Observation of the referees was performed in May and June 2021. Each referee was observed twice and scored using the R-PIASS tool [35]. The referee’s verbal and non-verbal communication was recorded using a GoPro Hero 8 camera and an Ejeas Fbim wireless intercom system. During the match, thanks to the wireless intercom, the observer could hear all the verbal messages of the referee. The observer took notes on all indicators of the dimensions of *building a positive climate* and *responsiveness to the needs of the players*. After the match, the observer compared observations and notes with the instructions for determining the scores in the R-PIASS tool, and, by analyzing the video once again, they rated the referee on each dimension. Next, the results were pseudonymized, transferred to a bulk Excel file, and stored securely in the MS Office cloud. The research was approved by the Senate’s Research Bioethics Commission of the Józef Piłsudski University of Physical Education in Warsaw, Poland (SKE 01–10–2020). The referees were informed about the assumptions and purpose of the study before the observation. Each referee voluntarily agreed to participate in the study by signing the consent form.

### 2.4. Analytic Strategy

Basic statistics such as percentages, arithmetic means, medians, and standard deviation were used to describe the data. A Shapiro–Wilk test, skewness, and kurtosis were used to examine if variables were normally distributed. A non-parametric Wilcoxon signed-rank test for two dependent samples was used to examine differences between referees’ ratings on two dimensions. The non-parametric Mann–Whitney U test was employed to test the significance of differences in the mean scores of referees between groups divided by experience. The level of significance was set at 0.05. The reliability of the tool was tested by Cronbach’s alpha coefficient. ICC estimates and their 95% confidence intervals were calculated based on a single-measurement (k = 2), absolute-agreement, 2-way mixed-effects model. Calculations were performed using the PASW Statistic 18 software (IBM Corp., Armonk, NY, USA).

## 3. Results

Individual indicators of the *positive climate* and *responsiveness* dimensions were rated on a three-point scale: Poor, average, and good. Scores for referee interactions on these two dimensions are presented in Table 4. When observing the first dimension, referees were rated poor in three out of four indicators. Nearly 70% of respondents received the lowest ratings. These results imply that most referees do not show interest in the players. They move around the pitch away from them and are not in physical proximity with them. Referees are usually not smiling and enthusiastic. They rarely give players positive comments. Only 13% of the referees show an attitude of actual interest in the players, thus building a positive climate during the game. The exception to this is the indicator of *mutual respect*. Here, the ratings were better, as half of the referees received top marks and the other half received average marks. High ratings mean that the referee and players are always respectful of each other. The players do not challenge the referee’s decision and recognize his or her authority. Referees use respectful language, such as “please,” “thank you,” and “you’re welcome,” and often refer to players by name, using a warm and calm tone of voice.

The referees’ *responsiveness to the players’ needs* during the game was assessed from the standpoint of three indicators: 1. Active monitoring of players’ emotional, cognitive, social, and health needs. 2. Responding to the players’ needs (fast meeting of the players’ needs). 3. Solving problems. Referees were rated highest in responding quickly to the players’ needs (52.2%) and solving problems (52.2%). Relating this result to the R-PIASS tool, this means that more than half of the referees were consistently responding to educational, social, emotional, and health needs. Highly rated referees provided adequate support to the players so that observed problems did not escalate and solutions were not postponed. Consequently, children in the care of more than half of the respondents felt comfortable and could always count on the support of a referee. It is noteworthy that the other half of the referees observed were not actively monitoring the needs of the children, and failed to anticipate problems. Referees rated low in this aspect did not move near players in search of signs indicating the need for additional support. They only responded when players clearly attempted to draw attention to themselves or when coaches, parents (fans), or teammates did this. Low or average referees’ ratings mean that the support provided was late.

**Figure 1:**
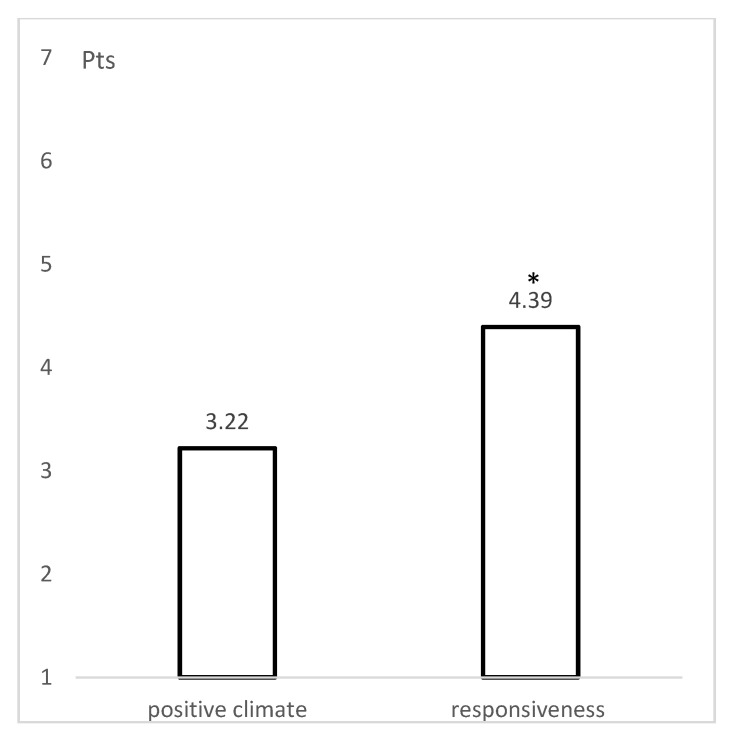
Mean ratings of the building positive climate and responsiveness to the players’ needs by referees (*n* = 23) during the match. * Significantly (*p* < 0.05) higher than the positive climate dimension. Pts.—points.

Based on the assessments of individual indicators, an evaluation of the referee’s work in the dimensions of *building a positive climate* and *responsiveness* was determined. Using the R-PIASS tool, these dimensions were scored on a 7-point scale (1, 2 means poor; 3, 4, 5 is average; and 6, 7 is a good rating). The results are presented in Figure 1. Referees were rated at an average level in both dimensions, 3.22 (SD = 1.65) and 4.39 (SD = 1.67), respectively, with the *responsiveness* dimension rating being statistically significantly higher than the *positive climate* rating (Z = −2.994; *p* = 0.003).

The study sought to determine whether referee’s ratings of the quality of their interactions on the dimensions studied were influenced by their refereeing experience. The results of the tests (Table 5) showed that referee experience was not a determinant of the rating of the quality of referee–player interactions in terms of *building a positive climate* on the field (Z = −0.068; *p* > 0.05) and *referee responsiveness to players’ needs* (Z = −0.165; *p* > 0.05). The Cronbach’s alpha coefficient from the analysis is 0.832, which indicates that the items have relatively high internal consistency. ICC (*positive climate*) was 0.801 with a 95% confidence interval from 0.576 to 0.912. ICC for the *responsiveness* dimension was 0.856 with a 95% confident interval of the ICC estimate of 0.694–0.936. The level of reliability was “moderate” to “excellent.”

## 4. Discussion

Based on a study by Pianta, Hamre, and Mintz [41], it was assumed that educational interactions between adults and children in sport can be divided into three domains: Emotional support, instructional support, and organizational support. The main aim of the study was to find out to what extent referees build a positive climate during the game and to what extent they respond to the needs of young players. These two dimensions show how the referee emotionally supports the children placed in his or her care. In school teacher–student relationships, there is already a well-established belief that the quality of interactions translates directly into better educational performance and frequency of prosocial behaviors. It is recognized that students perform better in challenging but supportive environments [6,41,42]. The same approach is transferred to sports. A study by Newton et al. [43] demonstrated that children’s experiences in sport are more positive when they perceive the surrounding climate as caring. Similar findings were presented by Fry and Gano-Overway [44] and Fry [45]. According to them, if a young athlete experiences emotional support in a club, he or she will be more likely to reciprocate such behavior toward his or her teammates, coaches, and referees. Other studies have shown that experiencing a positive climate in sports is linked to children’s ability to empathize, control their emotions, and exhibit social behavior [15]. For this reason, all adults involved in the organization of children’s sport are encouraged to make an effort to create a positive climate for the sporting event. Therefore, it is reasonable to assume that this postulate also applies to referees. In order for them to be able to cope with this task, they should have certain competencies. This research presents a list of referee behaviors that help build a positive climate, which is captured in four indicators: Emotional connection (physical proximity, social conversation, the players seek support from the referee); enthusiasm (smiling, engagement, positive affective reaction); positive comments (verbal and non-verbal); mutual respect (respectful and inclusive language, using players’ first names, calm voice, listening to players). The results presented above (Figure 1) indicate that referees do not always attach importance to this aspect of their work. Their influence on players was rated as average. This may result firstly from the programs of referee training courses, which usually focus on the rules of the game and their interpretation. Only then, with the next levels of professional excellence, are referees made aware of the need to build good relationships. However, the least experienced referees, who are often also very young, are mostly employed in children’s sport. A novice referee is concerned with controlling the rules and managing the game. The child’s world of needs is unknown to the referee. The most desirable situation would be to direct more experienced referees to children’s sport. At this point, it should be noted that the study did not positively verify the hypothesis (Table 5) that experienced referees have higher levels of interaction quality in both dimensions compared to inexperienced/novice referees. This hypothesis was based on the study by MacMahon et al. [46] and Dosseville, Laborde, and Garncarzyk [47], indicating that the referee’s quality of work increases with experience. Such a relationship was not confirmed in the present study. The explanation for such findings may be simple. The educational function of the referee, although desirable, is still not institutionalized and is not included in the curricula of referee training programs.

Another explanation for the average rating of referees in the dimension of *building a positive climate* is the widely acknowledged role of the referee on the field, with the referee rarely considered an educator. It is assumed that if anyone is to raise athletes, it is the coach and parent rather than the referee. This situation will change as the literature increasingly emphasizes the importance of the referee’s skills in building good relationships with players, communicating effectively, and managing the game and the players [48,49]. Lavay, French, and Henderson [7] pointed directly to the qualities that a physical activity specialist should have: Authenticity, enthusiasm, optimism, openness, and being congenial, all of which are preconditions for building a positive climate. A good referee should be characterized by passion for the sport, show respect for cultural and religious differences, have a sense of humor, and control his or her emotions. The average rating of the referees in the presented studies does not mean that they are bad professionals, but it indicates areas for improvement. The poor quality of referee–player interactions in terms of emotional connection (physical proximity, social conversation, the players seek support from the referee) should be confronted with the postulate that in addition to using the whistle, the referee should talk to the players [50]. The referee’s decisions are better accepted if the referee communicates them in a calm but confident and respectful voice [51,52]. The results of the evaluation of rugby referees are consistent with the observations of soccer referees, who were also rated according to the R-PIASS tool in the dimensions of *positive climate* and *responsiveness* at an average level (4.0) [53], and higher than for handball referees, rated at a low level (2.8) [35]. Interestingly, referees’ self-assessment of building a positive climate differs significantly from the results of their direct observation, e.g., football referees rated themselves highly on this dimension [54].

The emotional support of a young athlete also requires the ability to observe and recognize their emotional states [47]. In the opinion of referees, every good referee is characterized by the ability to interpret the players’ emotions and behaviors. This aspect of their work has been called *situation monitoring*, but it can also be described as reading players [48]. Although the rugby referees surveyed were admittedly rated significantly higher in the *responsiveness* dimension than in *building a positive climate* (Figure 1), it is still an average rating, which can be improved. Similarly, the *responsiveness* dimension was rated at the average level in other studies of handball and soccer referees [35,53]. With the dynamic situation on the sports field, the competitive atmosphere, and the pressure on a good performance, the referee has to be constantly alert. In addition to proactively diagnosing the emotional, cognitive, behavioral, social, and health needs of athletes [11,55], he or she should respond appropriately to the situation in order to resolve problems as quickly as possible and prevent them from escalating over time. To do this effectively, the referee must not only take the child’s perspective but also help the child take the perspective of others [11,56]. Since every match is different, the referee cannot use the same means, methods, and techniques of educational interactions all the time. Different situations on the pitch require the use of an appropriate refereeing style and matching it with the atmosphere of the match. This is particularly important in so-called invasion games, where the referee’s decisions cannot be based on strict enforcing the rules of the game [57,58,59]. Refereeing in this case requires creative and fair decisions that go beyond such rules [37]. The research was conducted on a group of 23 rugby referees, which is relatively small. This is a limitation when it comes to statistical inference, but at the same time, due to the small number of rugby referees in Poland, these are studies of almost the entire population and the results can be generalized. In the controlled observation method, participants are aware of being observed, which may influence their behaviors and is a limitation to be aware of. This does not reduce the observation value, because we know that the referees’ assessment reflected the maximum of their abilities. To date, studies have been conducted among referees in team sports, but there are more disciplines in which referees interact with players in numerous ways. It would be worth examining, among others, referees in martial arts and combat sports.

## 5. Conclusions

A lot of the power that referees are given in sports is associated with many responsibilities that change depending on the age of the players, the level of competition, and, above all, the expectations of the sports community, including parents. If sport is to serve the multifaceted development of children, one cannot fail to notice that the person responsible for this development is also the referee. Although the referee has a heavy workload, he or she must take on the additional responsibility of supporting the players emotionally. To put it simply, the referee should also become an educator and have an educational function on the pitch [60]. The educational function will further reinforce the reason why the referee should manage the game. If more and more children are to stay in the sport and not give up early, they have to be provided with good conditions. Efforts should be made to transform the sports field into a child-friendly educational environment. To date, referees’ efforts in this area have been based on a “tacit curriculum,” based on their own experiences and the advice of colleagues, sometimes experts. There have been few empirical studies to date describing the educational impact of referees on athletes. The present study also supports the postulate of institutionalizing the educational function of the referees. Performing this function effectively is difficult. Although the rules of the game and their interpretation are easy to learn by a referee, being a referee-educator requires a higher level of competence. The conclusions of the study are:

- There is a need to complement the existing training programs for referees with the contents from the field of pedagogy and developmental psychology. The indicators of emotional support identified in this research can be used here. They will help referees build a positive climate during the game and show how to respond to children’s health, cognitive, emotional, and social needs;
- improving a referee’s pedagogical skills can improve their relationship with players, which can ultimately translate into a greater sense of job satisfaction;
- the formulated practical implications can also be transposed to other team sports.

## Figures and Tables

**Table 1:** Descriptions of indicators [35].

Positive Climate			
Indicators	Poor (1,2)	Average (3,4,5)	Good (6,7)
Emotional connection - physical proximity - social conversation - the players seek support from the referee	Clear physical and emotional distance between the referee and players is observed. Apart from the messages related to the game, the referee does not talk to the players.	It can be observed that the referee and the players show mutual interest, but this only applies to one team or individual players. A physical and emotional distance between the referee and the players is sometimes observed.	The referee shows great interest in all players. Physical contact and emotional closeness are observed. Their relationship is warm and supportive. The referee sometimes talks to the players about problems unrelated to the game.
Enthusiasm - smiling - engagement - positive affective reaction	The referee does not show an enthusiastic attitude towards the players and his or her duties. They do not smile at all and do not reciprocate the positive emotions of the players.	The referee is enthusiastic and smiles, but there are moments when he or she does not do this or not to all players. The referee sometimes reciprocates the positive emotions of the players.	The referee shows enthusiastic attitudes and often smiles. He or she always reciprocates the positive emotions of the players.
Positive comments - verbal and non-verbal comments	The referee does not give positive comments to the players at all.	The referee sometimes gives positive comments to the players or does it often, but they are apparently insincere. The referee gives positive comments to only one team or selected players.	The referee often gives positive comments to all players and they are apparently sincere and unforced.
Mutual respect - respectful and inclusive language - using players’ first names - calm voice - listening to players	The referee and players rarely, if ever, demonstrate respect for one another. Competitors do not recognize the authority of the referee, often questioning his or her decision.	The referee and players sometimes demonstrate respect for one another; however, these interactions are not consistently observed across time or players and it happens that the players question the referee’s authority.	The referee and players consistently demonstrate respect for one another. The referee has the authority and his/her decisions are not called into question.
**Responsiveness**			
**Indicators**	**Poor (1,2)**	**Average (3,4,5)**	**Good (6,7)**
Active monitoring of players’ emotional, cognitive, social, and health needs	The referee does not monitor the players to meet their needs and does not know when the players need additional support or help.	The referee sometimes monitors the players to meet their needs and notices when they need extra support or help, but there are moments when this does not happen.	The referee constantly monitors the players to meet their needs and always notices when they need additional support or help.
Responding to the players’ needs - fast meeting of the players’ needs	The referee does not respond to or neglects the players’ needs.	The referee sometimes responds to the players’ needs, or this reaction does not apply to everyone.	The referee always responds to the social, emotional, and health needs of the players.
Solving problems	The referee cannot solve a problem that goes on and on.	The referee attempts to solve the problem, but he or she does not always do it effectively.	The referee manages to solve the problems that arise and they do not last long.

**Table 2:** Examples of low and high quality of referee–player interactions in the dimension of *building a positive climate*.

Indicators		Poor	Good
Emotional connection	Physical proximity	The referee stands in the middle of the field near the sideline, not following the action.	The player is limping. The referee runs up to him and pats him on the back to comfort him.
	Social conversation	The referee does not talk to the players about subjects unrelated to the match. When a player tries to talk to the referee before the game, the referee ignores or reproves him or her.	Before the match, the referee asks the players how their trip was and if they are feeling well today.
Enthusiasm	Smiling	The referee is poker-faced throughout the match and does not smile at the players.	During the match, the referee is smiling and exudes positive energy.
	Engagement	One can see that the referee does not care about the events on the field and would like this match to be over already. The referee avoids any interaction with the players.	The referee performs his duties with joy and energy. The referee is happy to interact with players when possible or needed.
	Positive affective reaction	As the players are happy after scoring a goal, the referee, with a stern face, tells them to stop and go back to the game.	The referee accidentally tripped on the field, which amused the players. The referee laughs along with them.
Positive comments	Verbal comments	The referee does not praise the players for their positive behavior.	The referee asks the defender to step back as the player is too close to the ball. When a competitor stepped back to the proper distance, the referee says: “Good job!”
	Non-verbal comments	The referee does not praise the players for their positive behavior.	The referee shows a gesture to a player to move back a few meters with the ball during a throw-in. When a player obeys the command, the referee praises him by raising his thumb up.
Mutual respect	Respectful and inclusive language	The player protests, to which the referee responds: “If you want to talk to me, we can meet for coffee later.”	The referee uses words such as “please,” “thank you,” and “excuse me.”
	Using players’ first names	The referee addresses the player: “Hey, you,” “you in the green shorts.”	During a penalty kick, the referee says to the goalkeeper: “Jacob, remember you have to stand on the goal line.”
	Calm voice	The referee is getting frustrated and is shouting at the players.	The referee, seeing the sad player, tells the player in a warm and calm voice not to worry, because in the next game they might be able to win.
	Listening to players	At halftime of a match, a player walks up to the referee and wants to ask about something, and the referee ignores him, saying he does not have time.	During a game, a player approaches the referee and reports that an opposing player is grabbing them by the jersey. The referee listens to him and says he would look into these situations.

**Table 3:** Examples of low- and high-quality interactions in referee’s *responsiveness to players’ needs*.

Indicators	Poor	Good
Active monitoring of players’ emotional, cognitive, social, and health needs	The referee failed to notice that one player was crying during the game.	Two players collided with their heads. Although the players did not report any problems, the referee ran up to them and asked if they were okay and could continue playing.
Responding to the players’ needs - fast meeting of the players’ needs	The player has twisted an ankle and is lying on the pitch. The referee paused the game only after comments from coaches and other players.	A player signals the problem with the turf to the referee. Without stopping the game, the referee quickly runs up to the player to find out what the problem is.
Solving problems	A player signals that an opponent is holding him down. The referee does not respond to repeated calls, the problem continues, escalates, and as a result, a frustrated player pushes his opponent.	A fan standing near the sideline interferes with the goalkeeper. The referee immediately pulls the fan away from the sideline.

**Table 4:** Assessment the quality of referee–player interactions in the dimension of *building a positive climate* and *responsiveness to players’ needs* during children’s rugby competition (*n* = 23).

		Quality of Referee–Players’ Interactions		
Dimension	Indicators	Poor	Average	Good
Building positive climate	Emotional connection (physical proximity, social conversation, the players seek support from the referee)	69.6%	17.4%	13.0%
	Enthusiasm (smiling, engagement, positive affective reaction)	69.6%	17.4%	13.0%
	Positive comments (verbal and non-verbal)	65.2%	21.7%	13.0%
	Mutual respect (respectful and inclusive language, using players first names, calm voice listening to players)	4.4%	43.5%	52.2%
Responsiveness to the players’ needs	Active monitoring of players’ emotional, cognitive, social, and health needs	47.8%	39.1%	13.0%
	Responding to the players’ needs (fast meeting of the players’ needs)	13.0%	34.8%	52.2%
	Solving problems	13.0%	34.8%	52.2%

**Table 5:** Differences in the assessment of the quality of referee–players’ interactions in all dimensions between experienced and inexperienced referees (*n* = 23).

Dimensions	Rugby Referees				Mann–Whitney U-test	
	Beginner (*n* = 15)		Experienced (*n* = 8)			
	Median	$\bar{x}$ Rang	Median	$\bar{x}$ Rang	Z	*p*
Building positive climate	3.00	11.93	4.00	12.13	−0.068	0.945
Responsiveness to the players’ needs	3.00	12.17	4.50	11.69	−0.165	0.869

## Data Availability

Not applicable.
